# Design, implementation, and assessment of an independent second-check system for proton and carbon-ion pencil-beam scanning: delivery-aware Monte Carlo and microdosimetric RBE-weighted dose

**DOI:** 10.3389/fonc.2026.1949825

**Published:** 2026-10-20

**Authors:** Jun Tan, Keith Furutani, Alessio Parisi, Shannon Hartzell, Chris Beltran

**Affiliations:** Department of Radiation Oncology, Mayo Clinic Florida, Jacksonville, FL, United States

**Keywords:** carbon-ion radiotherapy, delivery-aware Monte Carlo, independent second check, MCF MKM, microdosimetry, pencil-beam scanning (PBS), proton therapy, relative biological effectiveness

## Abstract

**Background:**

Independent second checks for proton and carbon-ion pencil-beam scanning should assess the planned physical dose, the expected delivery sequence, and the model-dependent relative biological effectiveness (RBE)-weighted dose.

**Objective:**

The aim of this study was to design, implement, and assess a traceable second-check system that combines delivery-aware Monte Carlo transport with microdosimetric RBE calculation for both particle species.

**Methods:**

The system receives Digital Imaging and Communications in Medicine (DICOM) planning data, converts the discrete spot sequence into a predicted delivery sequence for dose-driven continuous scanning (DDCS), generates weighted particle histories, and performs independent TOPAS/Geant4 transport. It scores physical dose, dose-averaged linear energy transfer (LETd), and voxel-level microdosimetric quantities. For both beam types, the scored lineal-energy distribution is represented using abridged microdosimetry distribution methodology (AMDM) and processed with the Mayo Clinic Florida microdosimetric kinetic model (MCF MKM). The proton branch additionally produces a fixed-RBE-weighted dose using RBE = 1.1. No fixed-RBE model is used for carbon ions. Proton assessment included component tests, single-energy source, transport, and scoring checks at 70.0, 170.2, and 229.1 MeV, patient-level evaluations using de-identified plans, and operational timing. Carbon-ion assessment used a pre-existing Monte Carlo dataset to evaluate component-level handling and visualization of AMDM descriptors, the MCF MKM RBE factor, and RBE-weighted dose.

**Results:**

The integrated workflow completed plan receiving, delivery-sequence generation, particle transport, physical and biological dose calculation, comparison, and report generation. The proton case for which detailed quantitative end-to-end results were reported was classified as REVIEW REQUIRED because not all dosimetric comparison criteria were met. A pre-existing carbon-ion dataset was used to verify the component-level handling and separate presentation of physical dose, AMDM descriptors, RBE factor, and RBE-weighted dose. Predicted DDCS timing and the stationary-versus-moving irradiation components were not benchmarked against machine logs or measurements.

**Conclusion:**

A common delivery-aware platform was implemented to support independent physical dose and microdosimetric RBE-weighted dose assessment for proton and carbon-ion treatments while retaining beam-specific dose conventions and biological model information. Further local commissioning and multicase validation are required before clinical use.

## 1 Introduction

Pencil-beam scanning (PBS) delivers proton and carbon-ion treatments as ordered sets of energy layers and weighted beam positions. An independent second calculation provides a separate test of the treatment-planning system (TPS), transferred plan, patient geometry, beam model, and dose interpretation. Monte Carlo (MC) transport is particularly valuable because it represents particle interactions and heterogeneous anatomy, but a clinically useful second check also requires measurement-based commissioning, machine quality assurance, version-controlled configuration, and traceable reporting (1–3).

MC transport is well suited to independent patient-specific verification because it can model particle interactions and dose deposition in heterogeneous anatomy using a calculation pathway separate from the TPS. The system developed in this study uses TOPAS (3, 4), which is based on Geant4 (5–7), for independent particle transport and dose calculation. Other fast independent engines, including FRED and MCsquare for proton therapy and FRoG for proton and carbon-ion beams, have demonstrated that patient-specific recalculation can be performed within clinically practical times (8–11). However, most plan-based recalculation methods begin with the nominal spot positions, energies, and weights exported by the TPS. They therefore test the dose associated with the planned spot list, but do not directly test how the delivery-control system translates that plan into a time-ordered irradiation sequence. Log-file-based MC can reconstruct the recorded delivery after irradiation, whereas a predictive delivery model can evaluate this plan-to-delivery translation prospectively before treatment (12). This distinction is important for dose-driven continuous scanning (DDCS). In DDCS, irradiation can continue during magnet motion, and the delivered sequence depends on scan path, spot map, beam current, spill management, energy transitions, rapid beam-off behavior, skip or break logic, and inter-spot dose. These mechanisms have been studied individually through scan-path optimization, beam-current evaluation, rapid beam-off and skip-spot testing, inter-spot quality assurance, and temporal characterization (13–17). A delivery-aware second check should combine those machine rules with independent particle transport so that the predicted delivery sequence and its dosimetric consequences are evaluated before treatment.

Relative biological effectiveness (RBE)-weighted dose verification is relevant to both beam species, although the reporting conventions differ. Proton therapy commonly uses a constant RBE factor of 1.1 for clinical dose reporting, while experimental and clinical evidence shows that proton RBE varies with radiation quality, dose, biological endpoint, and tissue response (18). The Mayo Clinic Florida microdosimetric kinetic model (MCF MKM) provides a microdosimetry-based method for estimating variable proton RBE, and related work has examined its relationship with phenomenological proton RBE models, LET-based approximations, and secondary-fragment contributions (19–21). A complete proton second check can therefore report the conventional 1.1-weighted dose and a variable-RBE-weighted dose.

Carbon-ion therapy also requires independent verification of physical dose and biological weighting. Clinical carbon-ion dose depends on the selected RBE model, beam quality, fractionation, reference-photon response, and implementation. Modified microdosimetric kinetic model and local effect model lineages use different biological formalisms and can produce clinically relevant differences (22–29). Unlike the proton branch, a constant RBE factor is not appropriate for carbon-ion output; the carbon branch in the present system therefore calculates only the MCF MKM-derived RBE factor and RBE-weighted dose.

The MCF MKM and abridged microdosimetry distribution methodology (AMDM) provide a common microdosimetric foundation for particle RBE calculation (30, 31). A carbon-ion pencil-beam (PB) implementation calculates physical dose and AMDM quantities on patient computed tomography (CT) data and has been validated against TOPAS MC for four anatomical sites (32). That prior study provides supporting evidence for the MCF MKM/AMDM methodology and the associated PB implementation. The PB-versus-TOPAS simulations and their case-level numerical results were not repeated in the present platform study. Independent reproduction of clinical MKM dose from microdosimetric distributions provides additional support for using these quantities in quality assurance (33). Together, these developments allow one second-check platform to share Digital Imaging and Communications in Medicine (DICOM) processing, geometry, comparison, and reporting while retaining species-specific delivery and RBE logic.

The purpose of this study was to describe the design, implementation, and technical assessment of an integrated second-check system for proton and carbon-ion PBS. The study objectives were to (1) establish a common, traceable software architecture; (2) implement a delivery-aware proton MC branch that calculates physical dose reported by TPS (either to medium or to water), microdosimetry, conventional RBE 1.1 dose, and MCF MKM variable-RBE dose; (3) integrate a carbon-ion MCF MKM/AMDM branch that calculates physical and MCF MKM RBE-weighted dose; and (4) define an assessment framework that distinguishes component verification, proton patient-level assessment, prior method-level evidence, local commissioning, and clinical validation.

## 2 Materials and methods

### 2.1 Study design and system requirements

The second-check system uses a shared computational backbone for both proton and carbon-ion beams. After common ingestion of the Radiotherapy (RT) Ion Plan, planning CT, structures, dose objects, calibration data, and configuration information, the plan is processed by the same delivery-aware DDCS conversion and source-generation modules. These shared modules generate the time-ordered source description that is read by the same custom TOPAS source interface for MC transport. The same TOPAS/Geant4 transport framework is then used for both beam species to calculate physical dose to medium, physical dose to water, dose-averaged linear energy transfer (LETd), and voxel-level microdosimetric quantities. Dose to water, $D_{w}$, and dose to medium, $D_{m}$, are produced and retained as separately labeled dose grids. Neither is obtained from the other by post-processing conversion. The platform supports either convention to match the corresponding TPS reporting convention.

The distinction between proton and carbon-ion calculations begins after this shared physical-transport stage. For proton beams, the system reports physical dose and two RBE-weighted dose objects: the conventional RBE-weighted dose using a fixed RBE of 1.1, and the MCF MKM variable-RBE-weighted dose. For carbon-ion beams, the system reports physical dose and the MCF MKM-derived RBE factor and RBE-weighted dose only. Thus, the beam species share the same delivery-aware conversion, source generation, MC transport, and comparison framework, while differing mainly in the biological interpretation of the scored quantities and in the final RBE-weighted dose objects that are reported.

The comparison, evidence-gating, and reporting layers are also shared. For both beam species, the system performs integrity and configuration checks, source and delivery summaries, physical-dose comparison with the TPS, RBE-weighted dose comparison with the TPS, and standard reporting metrics such as gamma analysis, dose–volume histogram (DVH), profiles, and range-based checks.

The second-check system was implemented on an institutional Linux high-performance computing (HPC) cluster running Red Hat Enterprise Linux 8.10 on x86–64 hardware, with job scheduling managed by SLURM 24.05.3. Patient-specific MC simulations were submitted as independent field-level jobs through SLURM. Each field requested 256 CPU cores and 1 TB RAM, and TOPAS was configured to use one thread per core. The workflow was written in Python 3.12.7 and uses pydicom 3.0.1, NumPy 2.1.2, SciPy 1.14.1, and Matplotlib 3.10.1 for DICOM handling, numerical processing, and result generation.

All MC calculations performed for the present study used TOPAS 4.0 compiled with Geant4 11.3.0. Proton calculations used the modular physics list of these TOPAS module names: g4em-standard_opt3, g4h-phy_QGSP_BIC_HP, g4decay, g4ion-binarycascade, g4h-elastic_HP, and g4stopping. The g4em-standard_opt3 uses the Urban model for charged-particle multiple scattering. Carbon-ion calculations were performed using TOPAS module names: g4em-standard_opt3, g4h-phy_QGSP_BIC_HP, g4decay, g4ion-liqmd, g4h-elastic_HP, and g4stopping. Note that the nuclear model used for proton beams is g4ion-binarycascade while carbon beams used g4ion-liqmd (34). The upper kinetic-energy limit of the electromagnetic physics tables was 5.2 GeV, the production range cut for transported particles was 0.05 mm and the maximum step length within the patient geometry was 1 mm as a practical spatial-accuracy vs. computation-time compromise (35, 36). Secondary electrons and positrons were not transported, and their energies were deposited locally and attributed to the parent charged particle. This is commonly used for millimeter-scale ion-beam dose calculations because secondary-electron ranges are generally short relative to the scoring voxels. It has been adopted in the RayStation proton MC engine and previously validated proton and carbon-ion MC implementations (37–39).

CT voxels were assigned to variable-density materials using the Schneider CT-to-material conversion and tabulated calibrations for Hounsfield unit (HU) value to mass density and electron density, which can be easily changed to other CT conversion or material assignment by swapping in other configuration files such as our institution-specific data under calibration. Voxels outside the external body were assigned to air. The DICOM-RT Ion interface is used to extract treatment plan information from DICOM files and build geometry for MC simulations (40). All second-check configuration files, treatment-machine tables, multi-energy extraction data, nozzle geometry definitions, DDCS conversion utilities, and post-processing scripts were maintained within the project environment under a dedicated second-check system folder, while shared system resources were used only for the Python runtime, SLURM, environment setup, and TOPAS executable.

### 2.2 Common architecture and data flow

The system begins with a set of DICOM files of one treatment plan exported from the TPS, including RT Ion Plan, planning CT, structure set, physical dose, and RBE-weighted dose when available. A run manifest records the de-identified case identifier, beam species, plan and dose identifiers, frame of reference, calculation-engine version, beam-model version, material-conversion table, RBE-model version, biological parameters, dose convention, random seeds, and comparison settings. Input checks verify DICOM identifiers, geometry, voxel dimensions, orientation, plan units, beam species, and the expected dose object before calculation.

After the input checks, the shared DDCS converter transforms the discrete spot sequence in the RT Ion Plan into a time-ordered representation of the expected continuous-scanning delivery. For each energy layer, the converter reads the planned spot positions, energies, and weights and applies the machine configuration for the selected beam species. It calculates the path and travel time between successive spot positions and, when the beam remains on during magnet motion, represents the planned irradiation as a combination of dose delivered during the transition and dose delivered after the beam reaches the planned position. The converter also accounts for beam intensity, spill availability, energy changes, monitor thresholds, and beam-off, break, or skip conditions. The resulting sequence contains stationary irradiation events, irradiation during magnet motion, energy-transition intervals, and beam-state changes in their expected delivery order. This conversion does not alter the clinical plan; it produces a predicted DDCS delivery sequence for independent recalculation.

The resulting delivery sequence is passed to the shared source-generation module, which creates time-ordered source records containing particle species, position, energy, time, event type, beam intensity, and statistical weight. The custom TOPAS source reader uses these records to generate weighted particle histories. The same TOPAS/Geant4 transport framework is used for proton and carbon-ion beams, with beam-species-specific machine data, beam models, and transport settings selected from the run configuration. MC transport through the patient or phantom geometry produces physical dose, LETd, and the quantities required for the subsequent microdosimetric calculations.

The workflow separates only at the biological-dose calculation stage. For proton beams, the system produces conventional RBE-weighted dose using a fixed RBE of 1.1 and the MCF MKM variable-RBE-weighted dose. For carbon-ion beams, the system calculates the required AMDM microdosimetric quantities and produces the MCF MKM variable RBE factor and RBE-weighted dose. Both particle species share the same CT and structure processing, coordinate transformation, dose-grid registration and resampling, dose-difference mapping, gamma analysis, DVH extraction, profile analysis, report generation, and provenance tracking. **Figure 1** summarizes the shared calculation backbone and the beam-specific biological-dose outputs.

**Figure 1:**
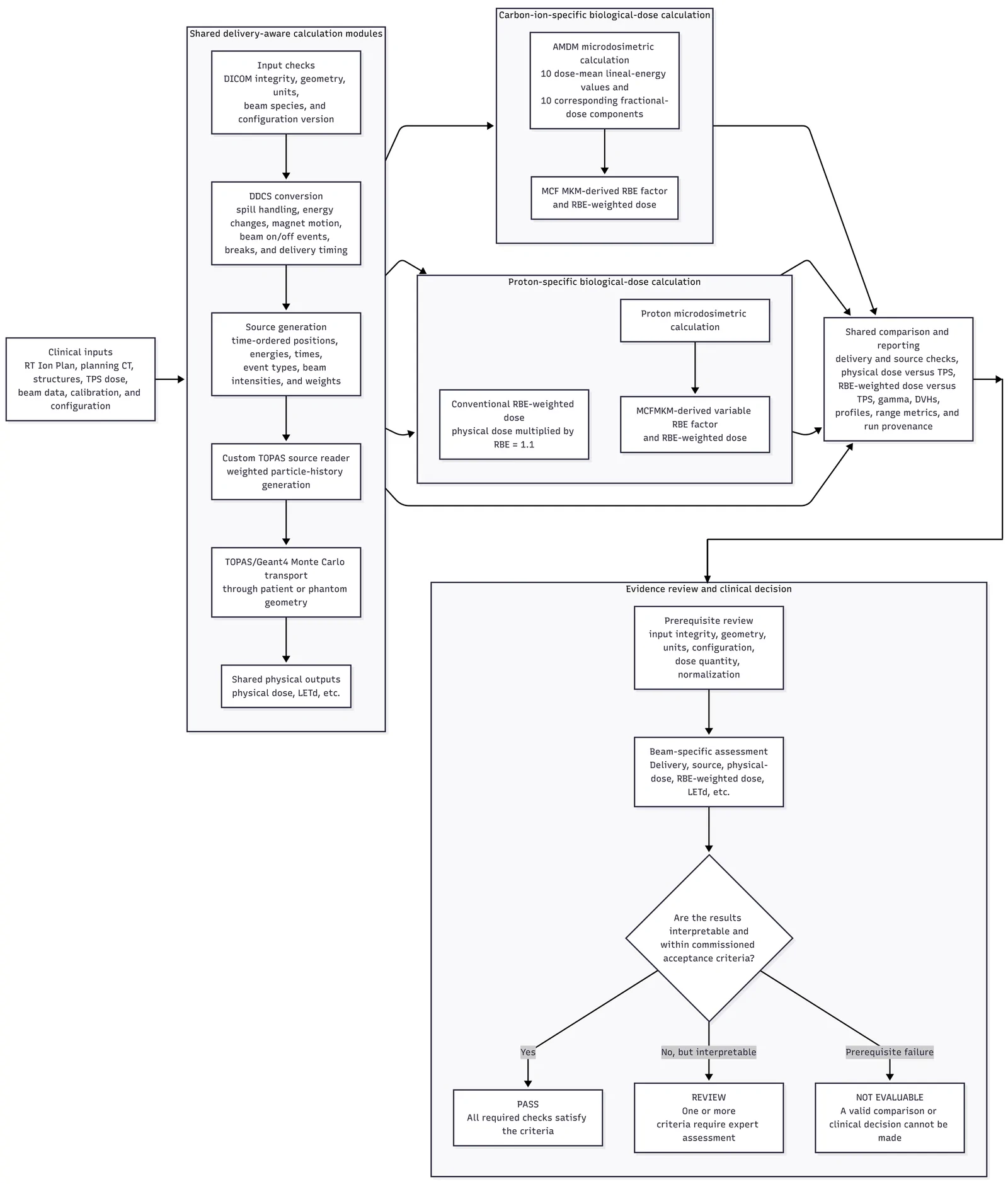
Architecture of the independent second-check system for proton and carbon-ion pencil-beam scanning.

The final report contains the physical dose, each available RBE-weighted dose, the associated RBE factor, LETd or microdosimetric quantities, comparison metrics, delivery diagnostics, calculation uncertainty, and all relevant model identifiers. Fixed-RBE and variable-RBE products are stored as separate dose objects and are never combined under an unlabeled term such as “biological dose”.

### 2.3 Microdosimetric scoring and MCF MKM RBE-weighted dose calculation

The common calculation architecture described in section 2.2 produced physical-dose and voxel-level microdosimetric outputs for both beam species. This section describes how those outputs were converted to voxelwise RBE and RBE-weighted dose with MCF MKM (30). Lineal energy y was defined as the energy imparted, ϵ, in a single energy deposition event divided by the mean chord length, $\bar{l}$, of the microscopic target.

(1) $$y=\frac{\epsilon }{\bar{l}}$$

The MCF MKM is a post-processing average implementation of the non-Poisson MKM that correlates clonogenic survival with microdosimetric distributions assessed in domains located within the cell nucleus. These domains represent structures such as giant chromatin loops or chromosome territories, where accumulated radiation-induced DNA damage may inactivate the domain and ultimately the cell. The model uses the lineal-energy distribution, the cell’s reference-photon response, and the mean radii of the nucleus ( $R_{n}$) and subnuclear domains ( $r_{d}$) to calculate the radiation-specific linear-quadratic coefficients α and β. Its non-Poisson correction describes overkill at high LET, while its radiation-quality-dependent calculation of β avoids the radiation-independent β assumption used in earlier MKM formulations. The resulting coefficients give $S(D)=exp(-\alpha D-\beta D^{2})$. The complete MCF MKM formalism is provided in the original publication (30).

For both proton and carbon-ion calculations, the scored lineal-energy distribution was represented using AMDM, which divides the distribution into 10 predefined lineal-energy intervals (31, 32). For bin *j*, the dose-mean lineal energy ${\bar{y}}_{D,\;bin\;j}$ and the corresponding dose fractions ${\text{Δ}}_{bin\;j}$ are defined in Equation 2, the same as in (31):

(2) $${\bar{y}}_{D,\;bin\;j}=\frac{\int_{y_{min,\;bin\;j}}^{y_{max,\;bin\;j}}yd(y)dy}{\int_{y_{min,\;bin\;j}}^{y_{max,\;bin\;j}}d(y)dy},\;{\text{Δ}}_{bin\;j}=\int_{y_{min,\;bin\;j}}^{y_{max,\;bin\;j}}d(y)dy$$

Here, d(y) is the density distribution of lineal energy *y*, and $y_{min,\;bin\;j}$ and $y_{max,\;bin\;j}$ are the lower and upper lineal-energy bounds of bin *j*, respectively. The AMDM assigns 21 physical quantities to each voxel v: the physical dose $D_{v}$, 10 values of ${\bar{y}}_{D,\;bin\;j}$, and 10 corresponding values of ${\text{Δ}}_{bin\;j}$. For all spot beams contributing to voxel i, the total effective AMDM parameters are obtained using the dose- and spectrum-weighted averages:

(3) $${\bar{y}}_{D,\;i,bin\;j}^{Total}=\frac{\sum_{k\in K}D_{i}^{k}\cdot {\text{Δ}}_{\;i,bin\;j}^{k}\cdot y_{D,\;i,bin\;j}^{k}}{\sum_{k\in K}D_{i}^{k}\cdot {\text{Δ}}_{\;i,bin\;j}^{k}},\;{\text{Δ}}_{\;i,bin\;j}^{Total}=\frac{\sum_{k\in K}D_{i}^{k}\cdot {\text{Δ}}_{\;i,bin\;j}^{k}}{\sum_{k\in K}D_{i}^{k}}$$

For the calculations reported in this study, $D_{i}^{k}$ in Equation 3 denotes the dose to water contributed by beam k to voxel i. For each bin *j*, ${\bar{y}}_{D,\;i,bin\;j}^{Total}$ is used to calculate the cell-specific linear term of the linear-quadratic model, α, and the correction factor c, using the AMDM equations reported in (32). The 10 bin values are then combined with the corresponding values of $\Delta_{\;i,bin\;j}^{Total}$ in Equation 4:

(4) $$\alpha =\sum_{j}\alpha_{bin\;j}\beta_{bin\;j},\;\beta =\beta_{ref}{\left(\sum_{j}c_{bin\;j}{\text{Δ}}_{bin\;j}\right)}^{2}$$

For proton beams, both the conventional RBE = 1.1 dose and the MCF MKM variable-RBE dose were calculated. The latter was included to independently evaluate the proton clinical dose-prescription system, which retains consistency with the conventional RBE = 1.1 target-dose prescription while accounting for spatial variations in radiation quality (41). An in-house-built TOPAS extension calculated the lineal-energy probability density distributions for primary and secondary ions. Secondary electron energy was assigned to the parent ion. The AMDM descriptors were derived from the scored lineal-energy distributions and the corresponding dose fractions.

For a chosen biological endpoint, biological RBE was defined as $D_{ref}/D$, the ratio between the absorbed doses required to obtain the same effect after exposure to the test and reference radiation. The calculations used the human salivary gland (HSG) parameter set listed in **Table 1**. The coefficients $\alpha_{ref}$ and $\beta_{ref}$ describe the linear-quadratic response to the corresponding reference radiation. Voxel RBE-weighted dose was calculated as (32):

**Table 1:** MCF MKM/AMDM parameters used in this study.

Parameter	Proton	Carbon ion	Unit
$\alpha_{0}$	0.089	0.089	${\text{Gy}}^{-1}$
$\beta_{0}$	0.0615	0.0615	${\text{Gy}}^{-2}$
$R_{n}$	4.64	4.64	μm
$r_{d}$	0.33	0.33	μm
$\alpha_{\text{ref}}$	0.228	0.787	${\text{Gy}}^{-1}$
$\beta_{\text{ref}}$	0.0606	0.0519	${\text{Gy}}^{-2}$
$F_{\text{clin}}$	1.1	2.41	Dimensionless

All listed parameters were used in the MCF MKM calculations, although not all appear explicitly in the equations presented in the main text. $\alpha_{0}$ and $\beta_{0}$ are the low-lineal-energy survival coefficients. $R_{n}$ and $r_{d}$ are the mean nuclear and subnuclear-domain radii, respectively.

$\alpha_{\text{ref}}$ and $\beta_{\text{ref}}$ describe the reference radiation response. $F_{\text{clin}}$ is the clinical scaling factor. AMDM represents the scored lineal-energy distribution using 10 dose-mean lineal-energy values and 10 corresponding dose fractions.

(5) $$D_{RBE}=RBE\times D_{phys}=\frac{\sqrt{\alpha_{ref^{2}}+4\beta_{ref}(\alpha D_{v^{w}}+\beta {\left(d_{v^{w}}\right)}^{2})}-\alpha_{ref}}{2\beta_{ref}D_{v^{w}}}\times D_{phys}$$

In Equation 5, $D_{v^{w}}$ is the dose-to-water input used in the MCF MKM biological-effect calculation, whereas $D_{phys}$ denotes the selected physical dose convention used for the reported RBE-weighted dose. For protons, $RBE_{bio}$ was defined as $D_{ref}/D$ for the same HSG surviving fraction, and $D_{clin}=D\cdot RBE_{clin}=D\cdot RBE_{bio}\cdot F_{clin}$, where $F_{clin}$ is the clinical scaling factor used to convert biological RBE to clinical RBE. $F_{clin}=1.1$ was used to maintain consistency with conventional proton clinical practice (36). The previously published carbon benchmark used OpenTOPAS 4.0 compiled with Geant4 11.1.3. Those simulations preceded the present platform study and were not repeated using the current TOPAS/Geant4 configuration. The MC approach explicitly simulated individual particle interactions, including nuclear fragmentation and secondary-particle generation (32).

### 2.4 Proton-specific RBE calculation and assessment

The proton workflow generates two RBE-weighted dose distributions using the $D_{phys}$ convention defined in Equation 5. For all proton results reported in the present study, $D_{phys}=D_{w}$, and this convention is applied consistently in the calculation, export, and TPS comparison. The fixed-RBE-weighted dose is defined in Equation 6:

(6) $$D_{RBE\;1.1}(v)=1.1\times D_{phys}(v)$$

and the variable-RBE-weighted dose is define in Equation 7

(7) $$D_{RBE,\;MCFMKM\;}(v)=RBE_{MCFMKM}(v)\times D_{phys}(v)$$

The physical dose, fixed-RBE dose, MCF MKM-derived RBE factor, and MCF MKM-derived RBE-weighted dose are retained as separate dose objects. Each output is labeled with its dose convention, RBE model, model version, biological parameter set, and fractionation conditions.

The fixed-RBE calculation is verified by confirming voxelwise agreement with $1.1\times D_{phys}(r)$ within numerical precision. The variable-RBE output was retained as a model-dependent result. Independent quantitative validation of the proton MCF MKM calculation was not performed in this study.

The fixed-RBE dose may be compared directly with the TPS clinical proton dose when the TPS uses the same RBE = 1.1 convention. A direct comparison of the variable-RBE dose requires the reference calculation to use the same MCF MKM version, biological parameters, dose convention, and fractionation. When these conditions are not met, the MCF MKM result is reported as an additional model-based assessment rather than as a pass-or-fail verification of the TPS dose.

### 2.5 Carbon-ion MCF MKM calculation and assessment

For carbon-ion beams, the microdosimetric information produced by the shared calculation stage is represented using the AMDM. AMDM stores 20 microdosimetric descriptors in each voxel: 10 dose-mean lineal-energy values and 10 corresponding fractional-dose contributions defined over predetermined lineal-energy intervals. Together with the physical dose, these descriptors provide 21 voxel-level quantities for the subsequent RBE calculation. This representation preserves information about the local microdosimetric distribution that would not be retained by a single LET-based quantity.

The MCF MKM uses the AMDM quantities, physical dose, dose per fraction, reference-photon response, and selected biological parameters to calculate the voxel-specific carbon-ion RBE factor. The corresponding RBE-weighted dose is calculated using Equation 5. The carbon-ion workflow provides only the MCF MKM-derived RBE factor and RBE-weighted dose. A fixed RBE value is not applied to carbon-ion dose. The physical dose, AMDM descriptors, RBE factor, and RBE-weighted dose are retained as separate outputs and are associated with the AMDM version, MCF MKM version, biological parameter set, fractionation, and dose convention used in the calculation.

The present carbon-ion assessment was limited to component-level handling, labeling, and visualization of the archived physical dose, AMDM descriptors, and RBE factor, together with the resulting dose-to-water RBE-weighted output.

A model-matched RBE comparison requires reference and second-check calculations to use the same MCF MKM version, biological parameters, fractionation, and dose convention. Comparisons with a TPS that uses a different biological model are treated as model-sensitivity assessments rather than direct validation of either RBE calculation.

The previously published carbon-ion benchmark in Ref (32). used dose to medium, $D_{m}$, for its final reported clinical RBE-weighted dose. The archived MC dataset contained $D_{w}$, the AMDM descriptors, and the MCF MKM RBE factor. These quantities were used to compute and display the corresponding dose-to-water RBE-weighted output without rerunning particle transport. Ref (32). is therefore cited as prior method-level evidence under its original reporting convention rather than as direct validation of the present dose-to-water output.

### 2.6 Verification tests and assessment endpoints

**Table 2** summarizes the assessment matrix, and **Table 3** summarizes the functional design and beam-species-specific outputs of the integrated second-check system. Common software tests covered DICOM parsing, coordinate transformation, material assignment, grid registration, resampling, DVH extraction, gamma implementation, report fields, and reproducibility. Gamma analysis followed the formulation of Low et al. (42). For the reported proton comparison, three-dimensional global gamma analysis was performed using the TPS dose as the reference distribution, a 3% dose-difference criterion normalized to the maximum TPS reference dose, and a 3-mm distance-to-agreement criterion. Voxels receiving less than 10% of the maximum TPS reference dose were excluded. The comparison dose was evaluated using trilinear interpolation. The gamma search used 0.15-mm increments and a maximum search radius of 6 mm.

**Table 2:** Assessment matrix and evidence sources for the independent second-check system.

Component	Assessment basis	Primary endpoints	Status and role in this study
Shared DICOM and data management	Synthetic objects and de-identified DICOM data.	Identifiers; frame of reference; geometry; units; branch selection; software and model versions; reproducibility.	Implemented and tested in both proton and carbon-ion branches.
DDCS models for proton and carbon-ion treatments	Deterministic spot and event tests; recording of requested and generated monitor units and source weights; timing decomposition; comparison with machine logs and measurements.	Spot and energy-layer order; identification and timing of move, stop, break, skip, and energy transition events; event-time accounting; predicted delivery time.	Component tests were implemented for the shared DDCS functions. The proton pathway was exercised end to end. A numerical source-weight residual was not retained for the reported patient run, and machine-specific tolerances must be established during local commissioning.
Proton Monte Carlo and physical dose calculation	Single-energy source, transport, and scoring checks at 70.0, 170.2, and 229.1 MeV; de-identified patient plans.	Source configuration; energy selection; transport completion; dose generation; dose convention; output labeling; patient-dose comparison	Functional and patient-level workflows were assessed. Physical beam-model commissioning requires local measurement data.
Proton biological dose calculation	Fixed RBE of 1.1; variable RBE calculated with MCF MKM.	RBE-weighted dose grid for RBE = 1.1; MCF MKM RBE factor; MCF MKM RBE-weighted dose; model version; biological parameter set; fractionation; dose convention.	Both RBE approaches were implemented and their outputs were labeled separately. Quantitative accuracy of the proton variable-RBE calculation was not evaluated in the study.
Carbon-ion microdosimetric and RBE-weighted dose outputs	Pre-existing carbon-ion Monte Carlo dataset; previously published PB-versus-TOPAS benchmark as supporting method-level evidence (32).	AMDM descriptor handling; consistency of the RBE factor and RBE-weighted dose; dose convention; output labeling; and visualization.	Assessed at the component level. No carbon-ion case was executed end to end in the present study.
Comparison and reporting	Independent reference dose distributions; known DVH values; gamma analysis regression cases; report-format tests.	Grid alignment; thresholds; masks; normalization; output labeling; data provenance; PASS/REVIEW decision rules.	A shared report framework was implemented for both beam types.

**Table 3:** Functional design and beam-species-specific outputs of the integrated second-check system.

Function	Proton branch	Carbon-ion branch
Plan and geometry	RT ion plan; planning CT; structures; physical dose; proton RBE-weighted dose using RBE = 1.1	RT ion plan; planning CT; structures; physical dose; carbon-ion RBE-weighted dose
Delivery sequence	Predicted DDCS delivery sequence, including planned spots and defined move, stop, break, skip, and energy-transition events.	
Independent calculation	Custom TOPAS source reader and Monte Carlo particle transport; MCF MKM/AMDM calculations of voxel-level dose-mean lineal energy and associated fractions.	
Dose and microdosimetric outputs	Physical dose; selected dose convention; LETd; 20 AMDM descriptors consisting of 10 dose-mean lineal-energy values and 10 corresponding dose fractions.	
RBE approaches	Fixed RBE of 1.1 and variable-RBE calculated with MCF MKM. The corresponding RBE-weighted dose grids are reported separately.	Variable RBE calculated with MCF MKM and the corresponding RBE-weighted dose. No fixed RBE is applied.
Comparisons	Physical dose; RBE-weighted dose for RBE = 1.1; variable RBE-weighted dose calculated with MCF MKM; gamma analysis; DVHs; dose profiles; range; delivery diagnostics; microdosimetric quantities; and computation time.	Physical dose; MCF MKM RBE factor and RBE-weighted dose; gamma analysis; DVHs; dose profiles; range; delivery diagnostics; AMDM quantities; computation time.

Proton component tests included exact parsing of spot positions, energies, and monitor units; preservation of spot order; deterministic handling of delivery events; independent timing checks; source-sampling reproducibility; and consistency between the physical-dose grid and the two RBE-weighted grids. Requested and generated source weights were recorded. The fixed-RBE test required the RBE 1.1 grid to equal 1.1 times the physical-dose grid at every voxel. Published proton microdosimetry and RBE studies were used to support the model description. The present implementation was not independently benchmarked against those studies.

Proton patient-level assessment included three de-identified PBS plans: a cranial plan with three fields, a head-and-neck plan with three fields, and a breast and regional-nodal plan with two fields. All three plans were used to exercise the patient-level second-check workflow, including plan ingestion, DDCS conversion, TOPAS transport, dose generation, post-processing, and report generation. Detailed quantitative dose comparison results are reported only for the breast and regional-nodal plan. The cranial and head-and-neck plans are reported as workflow completion and operational performance cases.

Carbon-ion assessment endpoints were limited to component-level handling, labeling, and visualization of the archived AMDM descriptors, RBE factor, and RBE-weighted dose. No carbon-ion case was executed end to end through the present platform.

MC sampling uncertainty for the three proton patient plans was evaluated in a separate repeat-seed study. Each of the eight fields was simulated six times at the full production history count using independent random number seeds. Per-field sample variance was calculated using an N−1 denominator, and plan-level variance was calculated as the sum of the field variances under the assumption of zero covariance between fields. Reported values represent one-standard-deviation Type A uncertainty for a single full-statistics calculation. Relative uncertainty was evaluated within the BODY contour using dose bands defined from the repeat-mean fixed-RBE clinical dose, absorbed dose to water multiplied by 1.1. Mean and 95th-percentile voxelwise uncertainties were reported. The voxelwise maximum was not used because of its sensitivity to isolated outlier voxels. No variance-reduction method was used. Uncertainty in the AMDM descriptors, MCF MKM RBE factor, and variable-RBE-weighted dose was not evaluated. Uncertainty in the primary target mean dose was calculated from the repeated plan-level mean-dose values.

### 2.7 Reporting and decision logic

The report is organized in the order in which the result should be interpreted: input integrity, calculation configuration, delivery or source summary, physical-dose comparison, biological-dose comparison, uncertainty, and final disposition. A failed prerequisite produces NOT EVALUABLE. An interpretable result that does not satisfy the configured criteria produces REVIEW. PASS, REVIEW, and NOT EVALUABLE are report dispositions under the configured research criteria and are not clinically commissioned action levels. A global gamma result is not used to override an error in beam species, dose units, model identity, geometry, or normalization.

No clinical action levels were derived in this study. The minimum report elements are summarized in **Figure 1**; **Table 2**, and requirements for locally commissioned tolerances are discussed in Section 4.4.

## 3 Results

### 3.1 Integrated system implementation

The common DICOM, DDCS, MC, comparison, and reporting modules were implemented. The complete workflow was exercised for proton cases. Carbon-ion assessment was limited to component-level processing of a pre-existing MC dataset. Each output retained its beam species, dose convention, RBE model, and parameter metadata.

For the completed second-check runs, each field was submitted as an independent MC calculation using 256 CPU cores and a requested memory allocation of 1 TB. Across eight completed field calculations from plans containing two or three fields, the recorded runtime ranged from 10.3 to 22.0 min per field, with a mean of 15.8 min. Because the fields were submitted independently and executed in parallel, the effective MC calculation time per plan was determined by the slowest field and ranged from 12.8 to 22.0 min. Peak resident memory use ranged from approximately 109 to 292 GB per field. All completed calculations proceeded through automated post-processing to generate the second-check DICOM dose and biological-dose outputs.

### 3.2 Proton branch implementation and assessment

The three proton plans completed the end-to-end workflow from RT Ion Plan ingestion through report generation and produced the physical, microdosimetric, fixed-RBE, and MCF MKM outputs summarized in **Table 4**.

**Table 4:** Proton outputs generated by the delivery-aware Monte Carlo branch.

Output	Definition	Independent comparison or use
Physical dose	Independently calculated dose, $D_{phys}$, reported as $D_{w}$ or $D_{m}$ according to the selected TPS convention.	Single-energy functional checks of source generation, transport, scoring, and output creation; patient comparison using dose profiles, DVHs, and gamma analysis.
RBE-weighted dose (RBE = 1.1)	$D_{RBE=1.1}=1.1\times D_{phys}$.	Comparison with the clinical TPS proton dose when the TPS uses the same fixed RBE.
MCF MKM RBE factor	Voxelwise RBE calculated from the AMDM descriptors, physical dose per fraction, reference radiation response, and specified biological parameters.	Comparison with a reference calculation using the same biological model and parameter set, fractionation, and dose convention; otherwise reported separately as a model-dependent assessment.
MCF MKM RBE-weighted dose	$D_{RBE,\;MCFMKM\;}=RBE_{MCFMKM}\times D_{phys}$.	Comparison using gamma analysis, DVHs, dose profiles, and region-specific dose differences when the biological model, parameter set, fractionation, and dose convention are matched.
LETd and microdosimetric quantities	Dose-averaged LET and microdosimetric quantities derived from particle transport and used as inputs to the MCF MKM calculation.	Comparison with reference depth profiles and spatial maps; verification of inputs to the biological model.
Delivery diagnostics	Spot map, stationary and moving irradiation; break and skip events; energy transitions; requested and generated source-weight summaries; predicted delivery time.	Deterministic component testing and report review; comparison with machine logs and measurements after local commissioning.

Single-energy calculations at 70.0, 170.2, and 229.1 MeV were used as functional checks of source generation, particle transport, dose scoring, and output generation. Quantitative agreement in range, distal falloff, and absolute output was not evaluated in this study.

The three plans comprised eight independent field-level MC calculations: three cranial fields, three head-and-neck fields, and two breast and regional-nodal fields. For the breast and regional-nodal plan, both field calculations completed successfully on SLURM using 256 TOPAS threads per field. Fields 1 and 2 simulated 42,684,965 and 40,585,241 primary histories in 18.2 and 21.5 min, respectively. DDCS preprocessing converted 20,644 and 19,564 planned spot entries into 21,343 and 20,293 time-ordered delivery-source entries, respectively. All generated control-point/spot-position (CP/SP) files conformed to the reference CP/SP schema, and the legacy input parser was not used. The final dosimetric comparison was classified as REVIEW REQUIRED. The mean dose difference in the high-dose region was −1.67%. Using a 3%/3 mm global gamma criterion and a 10% low-dose exclusion threshold, the gamma passing rate was 84.9%. Agreement of the DVH metrics varied by structure. All evaluated PTV SCV endpoints, D98, D95, D50, D2, and mean dose, met the report criteria, whereas D95 and/or D98 for PTV_IM and PTV_4256 triggered review^1^,^2^,^3^,^4^,^5^. Across the three proton plans, the mean one-run relative statistical uncertainty ranged from 0.79% to 1.14% in voxels receiving at least 90% of the prescription dose and from 1.00% to 1.62% in voxels receiving 50%–90% of the prescription dose. The corresponding 95th-percentile values ranged from 1.08% to 1.68% and from 1.49% to 2.57%, respectively. For the breast and regional-nodal plan, the mean and 95th-percentile uncertainties were 1.14% and 1.68% in the ≥90% dose region and 1.62% and 2.57% in the 50%–90% dose region. Detailed results are provided in **Table 5**. **Figure 2** presents representative spatial outputs for this proton case including dose to water, the fixed-RBE-weighted dose using RBE = 1.1, the MCF MKM RBE factor, the corresponding MCF MKM RBE-weighted dose, and the difference between the variable and fixed RBE-weighted dose distributions. Range difference, distal falloff, agreement between predicted and measured delivery time, stationary-versus-moving dose contributions, uncertainty in the MCF MKM RBE factor and variable-RBE-weighted dose, and quantitative MCF MKM validation were not evaluated. Therefore, no conclusions were drawn for those endpoints.

**Table 5:** Type A Monte Carlo statistical uncertainty for one full-statistics fixed-RBE proton dose.

Plan site	≥90% prescription dose, mean/95th percentile (%)	50%–90% prescription dose, mean/95th percentile (%)	Primary target $D_{mean}$ uncertainty (%)
Head-and-neck	0.86/1.27	1.07/1.86	0.013
Cranial	0.79/1.08	1.00/1.49	0.009
Breast and regional-nodal	1.14/1.68	1.62/2.57	0.007

Values are one-standard-deviation Type A uncertainties for the fixed-RBE proton clinical dose, $1.1\times D_{w\;}$. The 95th percentile is reported instead of the voxelwise maximum because the maximum is sensitive to isolated outlier voxels. These results do not include systematic uncertainty or uncertainty in the AMDM descriptors, MCF MKM RBE factor, or variable-RBE-weighted dose.

**Figure 2:**
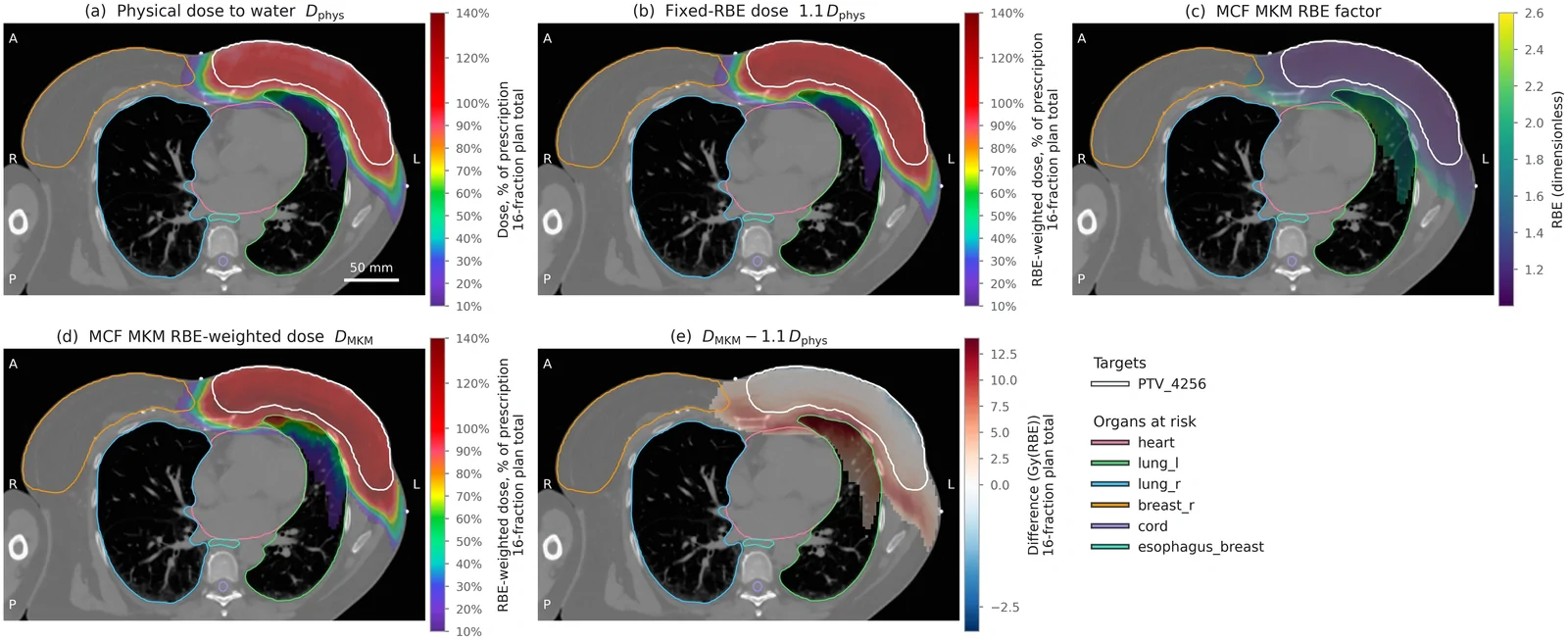
Representative proton dose outputs for the de-identified breast and regional-nodal plan, displayed on the same axial plane and spatial scale: **(a)** physical dose to water, $D_{phys}$; **(b)** fixed-RBE-weighted dose, $1.1\times D_{phys}$; **(c)** MCF MKM RBE factor; **(d)** MCF MKM RBE-weighted dose, $D_{MKM}$; and **(e)** signed difference, $D_{MKM}-1.1\times D_{phys}$. **(a, b, d)** are displayed as percentages of the prescribed 16-fraction plan dose. **(c)** is dimensionless. **(e)** is displayed in Gy(RBE). Target and selected organ-at-risk contours are overlaid. The RBE factor is displayed only within the specified dose mask.

The delivery-aware layer added traceable summaries of the spot map, stationary and moving irradiation, energy transitions, break or skip events, requested and generated source weights, and modeled delivery time. A numerical source-weight residual was not retained for the reported run. These outputs were linked to the same run manifest as the dose and RBE results, allowing a reviewer to determine whether a discrepancy arose from plan transfer, delivery modeling, particle transport, biological weighting, or comparison settings.

### 3.3 Carbon-ion branch implementation and assessment

A pre-existing carbon-ion MC dataset containing $D_{w}$, 20 AMDM descriptors, an MCF MKM RBE factor, and the corresponding dose-to-water RBE-weighted dose was used for component-level assessment of output processing and visualization. No carbon-ion case was tested end-to-end through the delivery-conversion, transport, provenance-recording, and reporting workflow in the present study. **Figure 3** shows a representative output set from this dataset.

**Figure 3:**
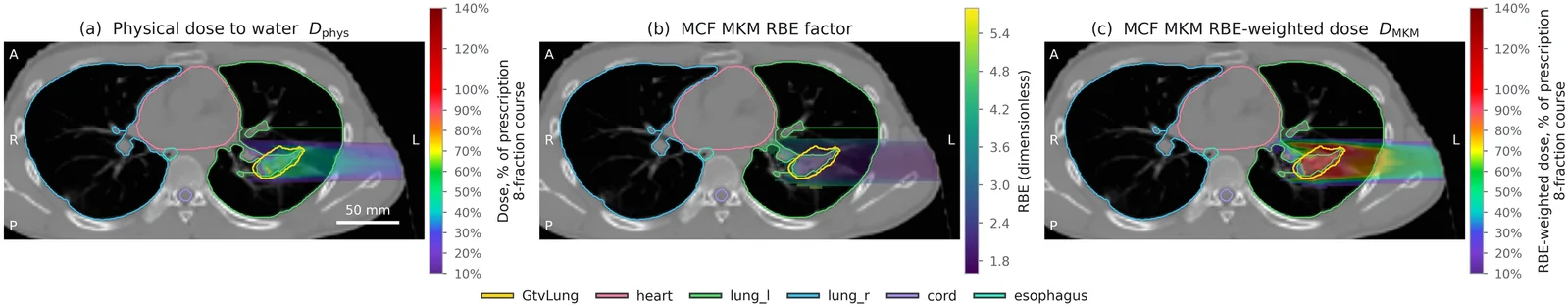
Representative carbon-ion quantities from a pre-existing Monte Carlo dataset, displayed on the same axial plane and spatial scale: **(a)** physical dose to water, $D_{phys}$, **(b)** MCF MKM RBE factor, and **(c)** MCF MKM RBE-weighted dose, $D_{MKM}$. **(a, c)** are displayed as percentages of prescription for the eight-fraction course, and **(b)** is dimensionless. Target and selected organ-at-risk contours are overlaid. The RBE factor is displayed only within the stated dose mask. The carbon-ion simulations were performed before assembly of the present platform and were not repeated in this study. The figure therefore demonstrates component-level processing and visualization, not end-to-end execution of the carbon-ion branch.

A carbon-ion PB implementation using the same AMDM/MCF MKM methodology was previously benchmarked against TOPAS MC using four clinical CT cases (32). Those simulations and their case-level gamma and computation-time results were not repeated or reproduced as results of the present study.

## 4 Discussion

### 4.1 Principal findings

The principal contribution of this study is the integration of delivery-sequence modeling, MC transport, and separately identified physical and biological-dose outputs within one second-check framework. The current evidence supports end-to-end technical assessment of the proton workflow and component-level assessment of the carbon-ion outputs, rather than clinical validation of either branch.

### 4.2 Role of delivery-aware Monte Carlo verification

Most plan-based second-check systems recalculate the nominal spot positions, energies, and weights exported by the TPS. This approach provides an independent test of the planned physical dose and patient model, and fast calculation engines have made it practical for patient-specific verification. However, the nominal spot list does not fully describe how a delivery-control system converts that plan into an ordered irradiation sequence.

The approach is prospective because it is generated from the plan and commissioned machine configuration before treatment. It is therefore different from log-file reconstruction, which uses recorded machine information after irradiation. The two approaches should be viewed as complementary. A prospective DDCS calculation can identify plan-to-delivery issues before treatment, whereas log-file reconstruction can test whether the actual irradiation followed the expected sequence. Agreement among the planned sequence, predicted DDCS sequence, and recorded delivery would provide stronger evidence than any one of these sources alone.

Delivery-aware calculation also introduces validation requirements that are not present in a nominal-spot recalculation. The converter must preserve the requested field weight while assigning appropriate weights to stationary irradiation, magnet-motion intervals, and beam-control events. Spot order, energy order, event timing, source position, statistical weight, and total delivered weight must therefore be tested explicitly. A correct MC transport calculation cannot compensate for an incorrect delivery sequence supplied to it. In this study, predicted DDCS timing and the stationary-versus-moving irradiation components were not compared with machine logs or controlled measurements and should therefore be regarded as implemented outputs rather than empirically validated delivery metrics.

The use of the same DDCS, source-generation, and TOPAS interface modules for proton and carbon-ion beams improves consistency, but it does not imply that the two delivery models are interchangeable. Scan speed, beam intensity, extraction behavior, particle calibration, energy switching, nozzle geometry, and other machine parameters remain beam-species specific. These parameters must be commissioned independently even when they are processed by the same software.

### 4.3 Microdosimetric and RBE-weighted dose verification

For protons, the fixed-RBE dose provides a deterministic scaling check and a model-matched comparison with a TPS dose calculated using RBE = 1.1. The MCF MKM output is a separate model-dependent assessment and should not be used as a pass-or-fail comparison unless the reference calculation uses the same model, parameters, fractionation, and dose convention. Quantitative validation of the proton variable-RBE calculation was not performed in this study.

For carbon ions, the MCF MKM result is the only RBE-weighted dose mode. Retaining physical dose, AMDM descriptors, RBE factor, and RBE-weighted dose as separate outputs supports interpretation and traceability.

### 4.4 Validation and assessment of the second-check system

Assessment of the second-check system should follow the calculation chain from software and data flow through delivery conversion, physical transport, and microdosimetric and RBE calculations. The present study addressed component testing and proton patient-level workflow assessment; benchmarking of predicted delivery timing against machine logs, local beam-model commissioning, and independent validation of the variable-RBE calculation remain outside the present scope.

The carbon-ion assessment was limited to component-level processing of a pre-existing MC dataset. No carbon-ion case was executed end to end through the present platform. Accordingly, the current carbon-ion results demonstrate compatibility of the AMDM and MCF MKM outputs with the shared processing framework rather than end-to-end validation of the carbon-ion branch.

Patient-level assessment should use several complementary endpoints. Gamma analysis is useful for summarizing spatial dose agreement but should not be the sole acceptance measure. DVHs, dose profiles, range differences, target and organ-at-risk metrics, LETd, microdosimetric quantities, RBE-factor differences, delivery timing, the residual between requested and generated source weight, and MC uncertainty provide different information about the calculation. Gamma analysis should be performed only after the dose quantity, units, normalization, coordinate system, orientation, and grid alignment have been confirmed.

The repeat-seed analysis showed approximately 0.8%–1.6% mean Type A uncertainty across the evaluated clinical-dose regions, with 95th-percentile values up to 2.6%. These estimates describe MC sampling uncertainty only and do not include systematic effects from CT calibration, stopping powers, patient positioning, machine modeling, dose-engine implementation, or biological-model parameters.

Clinical action levels should be derived prospectively from commissioning and validation data. Values reported in published studies cannot be transferred directly when the beam model, anatomy, calculation uncertainty, gamma implementation, dose threshold, or RBE parameters differ. The PASS, REVIEW, and NOT EVALUABLE categories are therefore most useful when each category is linked to a defined prerequisite and a locally validated tolerance rather than to a single universal gamma threshold. The REVIEW disposition cannot be attributed to a single mechanism from this case alone. Possible contributors include the absence of local beam-model commissioning, assumptions in beam model, CT-to-material conversion, comparison settings, and the heterogeneous and oblique breast and regional-nodal geometry. The contribution of the DDCS conversion could not be isolated because a matched nominal-spot control calculation was not included. A paired nominal-spot versus DDCS calculation, followed by comparison with machine logs or controlled measurements, is required in the next validation stage.

### 4.5 Clinical and operational implications

A shared computational framework reduces duplicated software while preserving the beam-model, physics, dose-convention, and biological-model information needed to interpret each second-check result. Shared software, however, can also create common-mode failures. An error in DICOM orientation, source normalization, material conversion, or dose-grid handling could affect both proton and carbon-ion calculations. Shared modules therefore require independent unit tests, reference datasets, change control, code review, and periodic regression testing. The fact that the second check is independent of the TPS itself does not guarantee that it is correct.

The HPC implementation allows the MC calculation to be parallelized by beam and particle history. Clinical practicality will nevertheless depend on more than the fastest individual calculation. Queue time, data transfer, failed-job recovery, reproducibility, report generation, and concurrent patient workload should be included in operational testing. A clinically useful system must return a traceable result within the required workflow time and must clearly indicate when a calculation is incomplete.

The system is intended to complement rather than replace existing machine QA, measurement-based commissioning, or post-delivery log review. Its main clinical role is to provide an independent, patient-specific assessment that combines the predicted delivery sequence with physical and RBE-weighted dose calculation before treatment.

The validation set should be expanded to cover the full clinical range of energies, field sizes, range shifters, heterogeneities, anatomical sites, fractionation schedules, and beam arrangements. Attention should be given to conditions with strong density changes, complex nuclear-fragmentation patterns, small fields, and high-LET regions. The carbon-ion benchmark should be extended with local machine data, and the proton MCF MKM calculation should be evaluated across a representative patient cohort rather than only selected examples.

## 5 Conclusion

An independent second-check system was implemented and technically assessed for delivery-aware particle transport and microdosimetric RBE-weighted dose calculation. Proton cases exercised the end-to-end calculation and reporting pathway, while a pre-existing carbon-ion MC dataset supported component-level assessment of AMDM descriptors, the MCF MKM RBE factor, and RBE-weighted dose. Together, these results demonstrate the feasibility of a shared, traceable framework for proton and carbon-ion second checks. Further local commissioning, end-to-end carbon-ion assessment, delivery-model benchmarking, and multicase validation are required before clinical use.

## Data Availability

The original contributions presented in the study are included in the article/supplementary material. Further inquiries can be directed to the corresponding author.

## Footnotes

1 Several dosimetric quantities are introduced in this sentence for the plan evaluated in this study.

2 1. PTV SCV: Planning target volume for the supraclavicular region.

3 2. PTV_IM: Planning target volume for internal mammary nodes region.

4 3. PTV_4256: Planning target volume defined by the 4,256-cGy isodose contour.

5 4. D98, D95, D50, and D2: The doses received by at least 98%, 95%, 50%, and 2% of a structure, respectively.
